# Vesicular Stomatitis Virus-Vectored Multi-Antigen Tuberculosis Vaccine Limits Bacterial Proliferation in Mice following a Single Intranasal Dose

**DOI:** 10.3389/fcimb.2017.00034

**Published:** 2017-02-07

**Authors:** Ming Zhang, Chunsheng Dong, Sidong Xiong

**Affiliations:** Jiangsu Key Laboratory of Infection and Immunity Institutes of Biology and Medical Sciences, Soochow UniversitySuzhou, China

**Keywords:** vesicular stomatitis virus, mycobacteria, tuberculosis, vaccine

## Abstract

Tuberculosis (TB) remains a serious health problem worldwide, and an urgent need exists to improve or replace the available vaccine, *Mycobacterium bovis* bacillus Calmette-Guérin (BCG). Most vaccination protocols adapt two or three doses to induce long-term lasting immunity. Our previous study showed that the naked DNA encoding the triple-antigen fusion TFP846 (Rv3615c-Mtb10.4-Rv2660c) induced robust T cellular immune responses accompanying four inoculations against mycobacteria infection. However, a number of compliance issues exist in some areas lacking the appropriate medical infrastructure with multiple administrations. In this study, a novel vesicular stomatitis virus expressing TFP846 (VSV-846) was developed and the immune responses elicited by VSV-846 were evaluated. We observed that intranasal delivery of VSV-846 induced a potent antigen-specific T cell response following a single dose and VSV-846 efficiently controlled bacterial growth to levels ~10-fold lower than that observed in the mock group 6 weeks post-infection in BCG-infected mice. Importantly, mice immunized with VSV-846 provided long-term protection against mycobacteria infection compared with those receiving p846 or BCG immunization. Increased memory T cells were also observed in the spleens of VSV-846-vaccinated mice, which could be a potential mechanism associated with long-term protective immune response. These findings supported the use of VSV as an antigen delivery vector with the potential for TB vaccine development.

## Introduction

Tuberculosis (TB) remains a serious health problem worldwide. One-third of the population has been infected with *Mycobacterium tuberculosis* (M.tb), and people infected with dormant M.tb are diagnosed as having non-clinical TB or latent TB infection, with ~5–10% of these people developing TB disease in their lives(WHO, 2004). Furthermore, latently infected individuals have an ~10%/year risk of developing active TB disease in HIV-infected persons if they do not receive antiretroviral therapy (Cohn, 2000). Therefore, successful vaccines against M.tb infection are needed.

*Mycobacterium bovis* bacillus Calmette-Guérin (BCG), the only available vaccine against TB, has failed to control adult TB and the establishment of latent persistent (TB) infection (Brewer, 2000; Glyn Hewinson, 2005). According to the previous studies, T cell-mediated immunity characterized by the secretion of IFN-γ and other cytokines plays an important role for the protection against M.tb infection (Cooper, 2009). Vaccine-specific T cell memory can also confer protection upon secondary challenge by a qualitatively different and quantitatively enhanced response (Henao-Tamayo et al., 2014).

We previously showed that a novel triple-antigen fusion DNA vaccine p846, engineered with three well-defined mycobacterial antigens [Rv3615c (Millington et al., 2011), Mtb10.4 (Hogarth et al., 2006), and Rv2660c (Betts et al., 2002)], induced robust T cell-mediated immune responses following four immunizations (Kong et al., 2014). Considering that there are many issues in some areas lacking the medical infrastructure to support multiple injections (Fachado et al., 2003), vaccination with a needle-free single dose should be considered as an important goal for vaccine development. Vesicular stomatitis virus (VSV), a negative-stranded RNA virus, has been proved to be a favorite viral vector for delivery of foreign vaccine antigens (Rose et al., 2001; Haglund et al., 2002; Clarke et al., 2007; Braxton et al., 2010). VSV has three major advantages as a potential delivery platform against TB. First, we and other groups demonstrated that VSV can be vaccinated through mucosa (Tan et al., 2005; Wu et al., 2014). It is believed that it is superior to elicit protective immune responses against infectious diseases at the site of infection (Davis, 2001). Second, with the help of the VSV reverse genetic system, additional genes can be easily inserted into the viral genome, and the recombinant VSV (rVSV) expressing foreign antigens can be grown to high titers in mammalian cell lines(Goonetilleke et al., 2003). Third, rVSV could stimulate potent humoral and cell-mediated immune responses in a needle-free single dose in animal models (Johnson et al., 1997; Faber et al., 2005; Cobleigh et al., 2010). Additionally, the prevalence of anti-VSV antibodies in the general population is extremely low. Therefore, few people carry pre-existing antibodies to counteract the VSV-based vaccines (Lichty et al., 2004). Previously, a VSV based TB vaccine VSVAg85A expressing the highly immunogenic antigen Ag85A was shown to be protective upon pulmonary M.tb challenge when administrated to mice intranasally (Roediger et al., 2008). However, this protection manifested at an early time-point as detected 2 weeks following immunization and was not sustained.

Here, we developed a VSV-based vaccine VSV-846 expressing a well-defined triple-antigen fusion gene that was effective against the mycobacterium infection described in our previous report (Kong et al., 2014). Our results indicated that vaccination with VSV-846 elicited a remarkable T cell-mediated immune response and provides effective long-term protection following BCG challenge within a needle-free single dose. These findings may also provide insight for further M.tb-vaccine development.

## Materials and methods

### Immunization protocols

Female BALB/c mice, 6–8 weeks old, were purchased from the experimental animal center of the Chinese Academy of Sciences and maintained in pathogen-free conditions. All animal experiments were performed in accordance with the laboratory animals' guidelines of the Laboratory Animal Ethical Commission of Soochow University (SYXK2014-0030).

Mice were lightly anesthetized with 30% isoflurane (Baxter) diluted in propylene glycol prior to all immunizations. Single intranasal inoculations of 10^6^ PFU were administered in a 25 μl volume for VSV-846. For BCG immunization, mice received a single dose of 1 × 10^7^ colony-forming units (CFU) BCG subcutaneously. For plasmid immunization, mice were intramuscularly injected with 50 μg p846 for 4 times biweekly. (1) For VSV-846 induced immune response and protection detection, each group consisted of twelve mice. Six mice per group were sacrificed 2 weeks after the final DNA vaccination for IFN-γ release assay, Lymphocyte proliferation assay and cytotoxic T lymphocyte measument. The other six mice per group were challenged with BCG 6 weeks after the final DNA vaccination and sacrificed 6 weeks after the challenge for bacterial load detection. (2) For VSV-846 induced long-term protection detection, each group consisted of eighteen mice. Six mice per group were sacrificed 6, 12, or 24 weeks after BCG challenge for the bacterial load detection and evaluation of pathology. (3) For VSV-846 induced memory T cells detection, each group consisted of six mice. These mice were sacrificed 24 weeks after the final DNA vaccination for memory T cells detection.

### Bacterial strains and culture conditions

*Escherichia coli* strain DH5α was grown in a Luria-Bertani medium for cloning. *M. bovis* BCG (Denmark strain 1331) was provided by the Center for Disease Control of Suzhou and was cultivated in a Middlebrook 7H9 medium or enumerated on 7H11 agar supplemented with 10% oleic acid-albumin-dextrose-catalase, 0.5% glycerol, and 0.05% Tween 80. Inactivated M.tb H37Rv strain was provided by the Fifth People's Hospital of Suzhou.

### Generation of VSV-based vaccine, VSV-846

The fusion gene-encoding Rv3615c, Mtb10.4, and Rv2660c was amplified by PCR as previously described (Kong et al., 2014). The PCR product was cleaved with XhoI and NheI and cloned into the fifth position of the pVSV-XN2 plasmid (Cobleigh et al., 2010), generating pVSV-XN_2_-846. The recombinant VSV-846 virus was generated by the following procedure: In brief, baby hamster kidney cells BHK-21 (ATCC number:CCL-10) grown to 60% confluence were infected with recombinant vaccinia virus expressing T7 RNA polymerase (Fuerst et al., 1986) and incubated for 1 h in serum-free Dulbecco's modified Eagle's medium (DMEM). Vaccinia virus-infected cells were then co-transfected with the plasmids pVSV-XN_2_-846 and the other plasmids for the expression of VSV N, P, and L (Ritz et al., 2012). Supernatants were collected 48 h post transfection, filtered through a 0.2 μm pore filter to remove vaccinia virus, and passaged onto fresh BHK-21 cells. The medium was collected immediately and filtered through a 0.2 μm pore filter after cytopathic effects were observed 2 days later. Recombinant VSV was then plaque purified and expanded. The titer was determined, and the VSV-846 was stored at −80°C until use.

### Western blot

BHK-21 cells were infected with VSV-846 and harvested 6 h post-infection. Cells were washed with phosphate-buffered saline (PBS) and lysed with 2 × SDS sample buffer. Proteins were separated on a 10% SDS gel, transferred to a nitrocellulose membrane, probed with anti-flag antibody (Sigma 1:2000), and detected with a secondary antibody (Southern Biotech 1:5000) using chemiluminescence.

### IFN-γ release assay

Two weeks after last immunization, spleen cells isolated from vaccine-immunized mice were plated at 5 × 10^6^ cells/well in 24-well plates. These cells were stimulated with TFP846 protein (10 μg/ml) as previously described (Kong et al., 2014) at 37°C for 72 h. The concentrations of IFN-γ in the culture supernatant were measured with an ELISA kit (eBioscience) according to the manufacturer's procedure.

### Lymphocyte proliferation assay

The proliferation of splenocytes from immunized mice was measured 2 weeks after the last immunization. Viable splenocytes were adjusted to a concentration of 5 × 10^6^ cells/ml and added to 96-well flat-bottomed plates at 5 × 10^5^ cells/well with 10 μg/ml of TFP846 protein. A BrdU-labeling reagent (Roche) was added to each well at a ratio of 1:1000. The culture plates were maintained in the same conditions for another 24 h and then incubated with anti-BrdU peroxidase. The absorption value at 370 nm was measured. Each sample was analyzed in triplicate.

### Measurement of cytotoxic T lymphocyte

Two weeks after the final DNA immunization, splenocytes were isolated and stimulated *in vitro* with 10 μg/mL of the recombinant TFP846 proteins in the vaccinated mice at 37°C, 5% CO_2_. Mouse myeloma cell line SP2/0 cells from Cell bank of Chinese Academy of Science were pulsed with the inactivated M. tb H37Rv for 24 h as target cells. A nonradioactive cytotoxic T lymphocyte (CTL) assay was performed with a lactate dehydrogenase (LDH) cytotoxicity detection kit (Roche). The effector cells were titrated in U-bottom 96-well plates at effector–target cell ratios of 50:1, 25:1, and 12.5:1; 1 × 10^4^ target cells were then added. After incubation at 37°C for 72 h, 100 uL of cell supernatant per well was removed and transferred into corresponding wells of the 96-well plate. A reaction mixture (100 uL) was added to each well, which was incubated at room temperature for 30 min. The absorbance value at 492 nm was measured. The percentage cytotoxicity of CTL was calculated as follows:

$$\begin{array}{l} \text{Cytotoxicity }(\%)=[(\text{effector and target cell mixture}\\ \;-\text{effector cell control})-\text{low control}]/\\ \;(\text{high control}-\text{low control})\times 100\%; \end{array}$$

According to the protocol, high control was the maximum releasable LDH activity in the cells (50 uL cell culture medium, 50 uL untreated cells and 5 uL lysis buffer). Low control was the spontaneous LDH activity released from the untreated normal cells (50 uL cell culture medium, 50 uL untreated cells).

### Mice challenage and colony-forming units assay

Six weeks after final DNA vaccination, the immunized mice were intranasally challenged with 1 × 10^7^ CFU of BCG as previouly described (Kong et al., 2014; Song et al., 2015). The bacteria load in the spleens and lungs at indicated time points post-challenge was counted according to the colony-forming units of serial dilutions of tissue homogenates on a Middlebrook 7H11 medium in triplicate.

### Histopathological analysis and inflammation severity evaluation

For histopathological analysis, lungs from the immunized mice (*n* = 6 per group) were collected 24 weeks post BCG challenge. The tissues were sectioned and stained with hematoxylin and eosin. Five sections were made from each mouse in one experiment. Two sections from each mouse were then randomly selected and total twelve sections of each group were evaluated for inflammation scoring by two independent investigators in one experiment. Inflammation score was assessed as the percentage of inflammation area compared with the overall size of the tissue section, with the aid of a microscope eyepiece grid as previously reported (Fairweather et al., 2005; Liu et al., 2014).

### Flow cytometry

Twenty four weeks post final DNA immunization, the splenocytes were isolated from the immunized mice (*n* = 6 per group). CD4^+^ memory cells were stained with PerCP-Cy5.5-anti-mouse CD4 (Biolegend), FITC-anti-mouse CD44 (Biolegend), PE-anti-mouse CD62L (Biolegend), and APC-Cy7-anti-mouse CD25 (Biolegend). CD8^+^ memory cells were stained with PerCP-anti-mouse CD8 (Biolegend), PE-anti-mouse CD62L (Biolegend), and Pacific blue-anti-mouse CD45R (Biolegend) antibodies. The stained cells were fixed with Cytofix/Cytoperm Buffer™ (Becton Dickson) and then analyzed with a FACS Canto II flow cytometer with FACSDiva software.

### Statistical analysis

Statistical analyses were performed with GraphPad Prism. Data were from three separate experiments and given as mean and standard deviation. The data were statistically analyzed by two-tailed independent Student's *t*-test through SPSS 12.0. The level of statistical significance was set to *P* < 0.05.

## Results

### Generation and identification of VSV-846 carrying mycobacteria triple-antigen fusion

**Figure 1:**
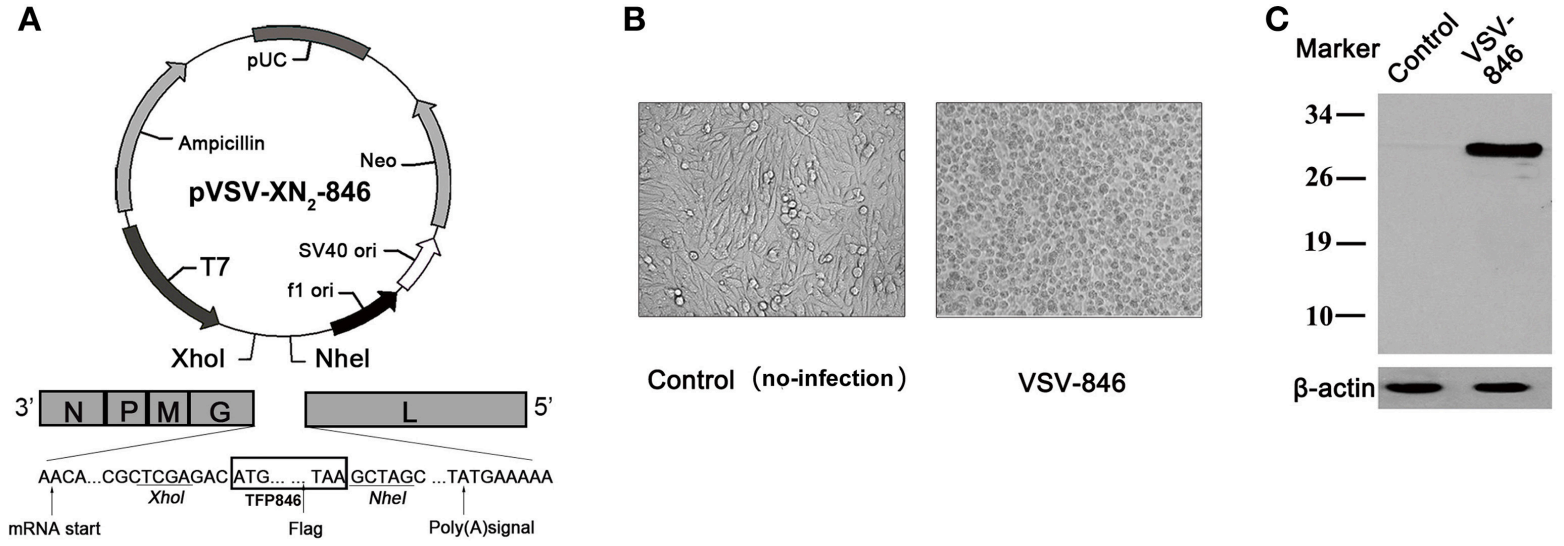
**Generation and identification of VSV-846 vaccine. (A)** The schematic of pVSV-XN2-846 plasmid. The triple-antigen fusion TFP846 (Rv3615c-Mtb10.4-Rv2660c) was amplified from p846 plasmid, and the triple-antigen fusion gene was then cloned into the fifth position of the pVSV-XN2 plasmid after cleavage with XhoI and NheI. **(B)** The microscope images of BHK-21 cells infected with VSV-846 or non-infected mock control 6 h post-infection (200×). **(C)** The triple-antigen fusion TFP846 was expressed in VSV-846 infected cells. The blot was probed with anti-flag antibody. Control: Normal cells without infection.

To generate VSV-based vaccine, the triple-antigen fusion TFP846 (Rv3615c-Mtb10.4-Rv2660c) gene in the p846 plasmid was amplified (Kong et al., 2014), and then cloned into the VSV genome at the fifth position of the VSV antigenome (Figure 1A). The rVSV in cell culture supernatant was harvested 48 h post transfection for purification. BHK-21 cells infected with rVSVs appeared cytopathic 6 h post-infection (Figure 1B). Expression of the triple-antigen gene was confirmed by anti-flag antibody (Figure 1C). These results indicated successfully generation of a VSV-based vaccine VSV-846 expressing M.tb triple-antigen fusion TFP846.

### Evaluation of VSV-846 induced cellular responses and protection

**Figure 2:**
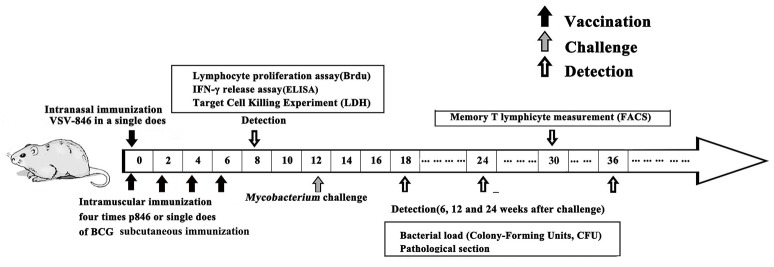
**Timeline of animal vaccination, challenge and detection**. Group of BALB/c mice was administered in a 25 μl volume for VSV-846 (single intranasal inoculations of 10^6^ PFU). Separately, mice immunized with 50 μg of p846 plasmid DNA administered intramuscularly or 10^6^ CFU BCG injected subcutaneously served as control groups. The immune response detection, BCG challenge, bacterial load, and evaluation of pathology were applied as indicated in the time points.

**Figure 3:**
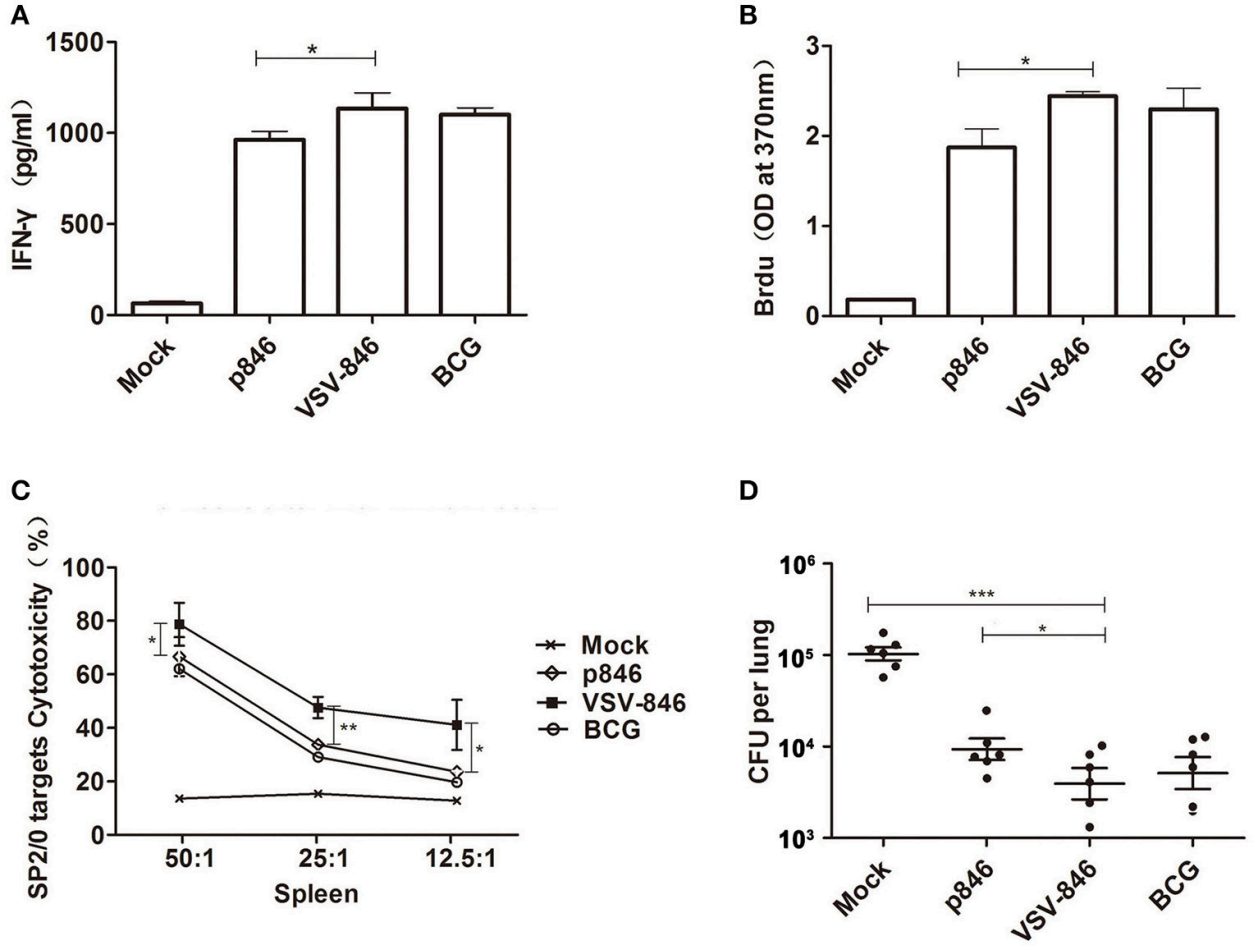
**Cellular responses and protection induced by VSV-846. (A)** Antigen-specific IFN-γ released from splenocytes purified from VSV-846-immunized BALB/c mice after *in vitro* stimulation with recombinant TFP846 2 weeks post immunization. **(B)** The specific T cells proliferation in spleen as measured by BrdU assay 2 weeks post immunization. **(C)** The specific cytotoxicity response was detected in the splenic tissue derived from mice immunized with VSV-846 at an E: T ratio of 50:1~12.5:1. For one experiment, each group consisted of six mice. Results are represented as the mean ± SD of three separate experiments. The error bar represents the standard deviation of three means in repeated experiments. ^*^p < 0.05, ^**^p < 0.01. **(D)** Bacterial numbers in one experiment (*n* = 6 per group) in the lungs of BALB/c mice subjected to various vaccinations challenged with *Mycobacterium bovis* BCG 6 weeks post vaccination. ^*^p < 0.05, ^***^p < 0.001.

Given the important role of cellular responses against mycobacteria infection, we evaluated the cellular responses induced by VSV-846 2 weeks after final DNA immunization (Figure 2). An IFN-γ release assay was performed to determine whether the VSV-846 vaccine was capable of inducing high levels of antigen specific IFN-γ^+^ T cells. Our results revealed that the level of IFN-γ secreted by VSV-846 immunized mouse spleen cells was higher compared with those from p846 immunized mouse spleens cells following incubation with TFP846 protein (Figure 3A, ^*^P < 0.05), and were similar to the level of IFN-γ secreted by BCG immunized mouse spleen cells. Significantly elevated antigen specific T cell proliferation was also observed in VSV-846 immunized mice in response to TFP846 protein, as compared with those of the mice immunized with p846 (Figure 3B, ^*^p < 0.05). Specific cytotoxic T lymphocyte (CTL) activity was assessed by using SP2/0 cells as target cells. The strongest antigen-specific cytotoxicity response was detected in VSV-846 immunized at an E:T ratio of 50:1 (Figure 3C, ^*^p < 0.05).

To investigate the degree of protection, immunized mice were intranasally challenged with 10^7^ colony-forming unit (CFU) BCG, and the bacterial loads in the lungs were determined 6 weeks post-infection. We observed that VSV-846 vaccination efficiently controlled bacterial growth to levels— ~10-fold lower than those observed in the mock group and 2.2-fold lower than those observed in the p846 group (Figure 3D, ^*^p < 0.05 and ^***^p < 0.001). However, the bacterial load in VSV-846 immunized mice was not significantly different from that observed in BCG immunized mice. These results showed that VSV-846 vaccination induced robust specific T cell immune response and protected mice from mycobacteria infection 12 weeks post-vaccination.

### VSV-846 immunization elicited long-term protection

**Figure 4:**
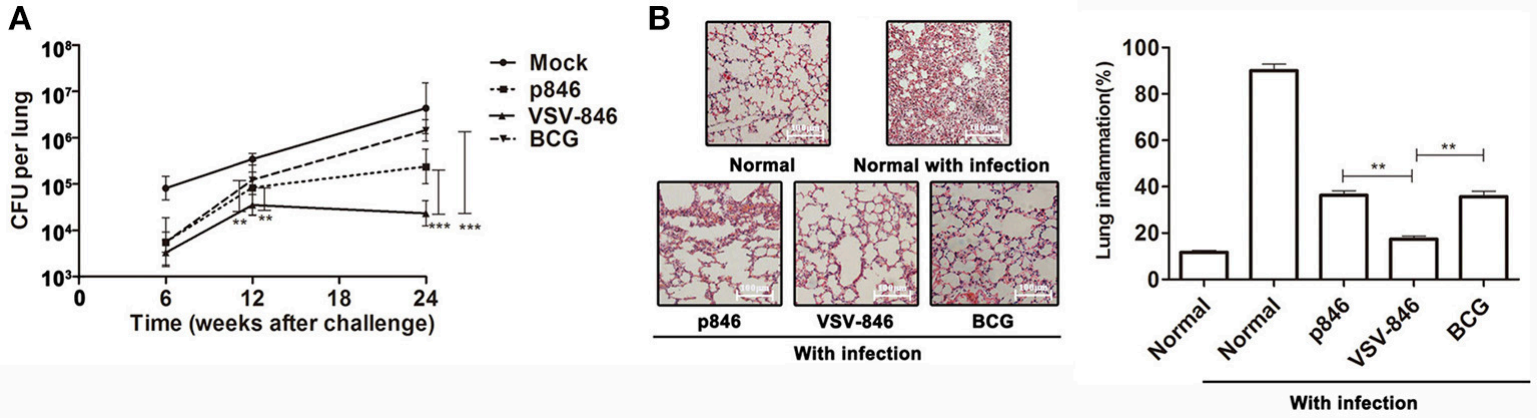
**Long-term immunity and protective efficacy of VSV-846. (A)** Groups of mice were immunized and challenged with *Mycobacterium bovis* BCG. The bacterial loads in mouse lungs were determined over a period of 24 weeks post-challenge. For one experiment, each group consisted of six mice. Results are represented as the mean ± SD of three separate experiments. The error bar represents the standard deviation of three means in repeated experiments. ^**^p < 0.01, ^***^p < 0.001. **(B)** Pathology of H&E-stained lung tissues (left panel, scale bar: 100 μm). and inflammation score (right panel) 24 weeks post-challenge are presented. Pictures shown are representative images of one experiment. Data of inflammation scores are represented as the mean ± SD of three separate experiments. The error bar represents the standard deviation of three means in repeated experiments. ^**^p < 0.01.

To determine whether VSV-846 vaccination induced long-term immune responses, groups of BALB/c mice were immunized and challenged. The bacterial loads in mouse lungs were evaluated over time. Consistent with results shown in Figure 3, 6 weeks post-bacterial challenge, mice were protected by VSV-846 vaccination. This method resulted in 1.47 log reduction of lung CFUs as compared with those observed in the mock control, although this finding was not significantly different from that of the BCG group. Interestingly, investigation of time points at 12 and 24 weeks post-challenge revealed that bacterial loads in the VSV-846-immunized group were significantly lower than those of the BCG group (Figure 4A, ^**^p < 0.01 and ^***^p < 0.001), suggesting that VSV-846 vaccination may induce better long-term protection against bacterial infection. Twenty four weeks after challenge, the pathology of lung tissues was analyzed. Sections from the normal mice were examined, revealing severe interstitial pneumonia, inflammation, and diffuse granuloma-like responses following BCG infection. However, VSV-846-immunized mice showed much less inflammation and intact alveolar morphology as compared with mice vaccinated with p846 or BCG, indicating that VSV-846 vaccination alleviated lung injury following BCG challenge (Figure 4B).

### VSV-846 increased the magnitude of memory CD4 ^+^ and CD8 ^+^T cells

**Figure 5:**
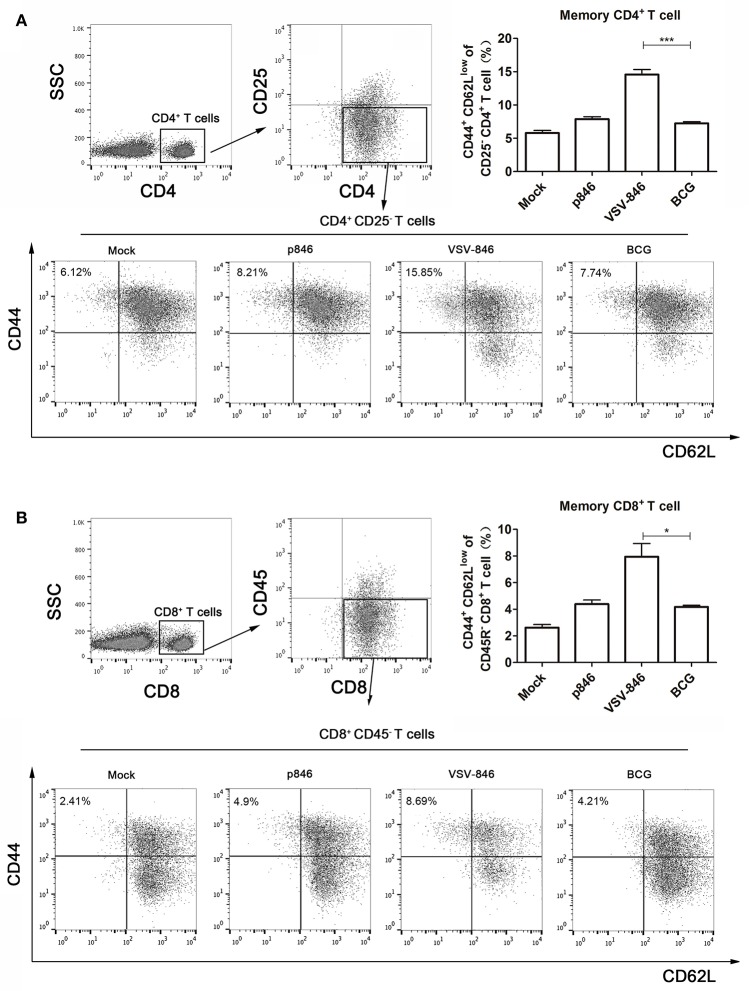
**Memory T cells immune responses induced by VSV-846 24-weeks post final DNA immunization, the splenocytes of immunized mice (*n* = 6 per group) were isolated and the memory T cells were analyzed by flow cytometry**. VSV-846 immunization particularly increased the frequency of memory **(A)** CD4^+^ and **(B)** CD8^+^ T cells compared with p846 or BCG immunization, suggesting that VSV-846 immunization may provide a more effective protection by inducing these T cells in the late stages of infection. The scatter plots are shown from the splenocytes of a single mouse. For one experiment, each group consisted of six mice. Results are represented as the mean ± SD of three separate experiments. The error bar represents the standard deviation of three means in repeated experiments. ^*^p < 0.05, ^***^p < 0.001.

Memory T cells are capable of responding more rapidly and mediating faster viral clearance upon re-exposure to antigen. A long-term immune response based on the presence of memory (CD44^+^ CD62L^Low^ CD25^−^) CD4^+^ T and (CD44^+^ CD62L^Low^ CD45R^−^) CD8^+^ Tcells is examined to determine protection from mycobacterium infection (Sondel et al., 2003). Mice were immunized with VSV-846, and 24 weeks after final DNA vaccination, splenocytes were isolated for quantifying memory T cell response. CD25^−^ T cells or CD45R^−^ T cells were gated from CD4^+^ or CD8^+^ T cells by flow cytometry, respectively. The long-term memory T cells were then characterized by CD44^+^ and CD62L^Low^. As shown in Figure 5, the percentage of both CD4^+^ (15.85%) and CD8^+^ (8.69%) memory T cells was higher in the VSV-846 group as compared with that observed in the BCG group (Figures 4A,B, ^*^p < 0.05 and ^***^p < 0.001), indicating that VSV-846 immunization was capable of providing sustained cellular immunity.

## Discussion

In recent years, immunization with live replication-competent VSV vaccines has been shown to be an effective method of vaccine development (Rose et al., 2001; Haglund et al., 2002; Clarke et al., 2007; Braxton et al., 2010). VSV vectors shared certain advantages for use in vaccine delivery. Specifically, they can induce robust humoral and cell-mediated Th1 immunity following a single dose in the absence of an additional adjuvant (Publicover et al., 2005). So far, traditional vaccinations require repeated doses to induce long-lasting immunity and specific adjuvant are often needed. For example, the two-dose HeVsG vaccine requires the action of two adjuvants, all hydrogels, and the CpG oligo deoxynucleotide (Bossart et al., 2012) to generate a Th1 immune response. However, hydrogels alone typically induce a Th2 response that is not appropriate against viral infections (Coffman et al., 2010; Steinhagen et al., 2011).

We previously reported that a naked DNA plasmid encoding TFP846 protein inoculated four times elicited a robust T cell-mediated immune response (Kong et al., 2014). In this study, we showed that protection along with long-term immune response against mycobacterial infection in mice was effectively achieved by intranasal immunization with a single-dose of VSV-based vaccine, VSV-846.

Vaccine induced CD4^+^ T cells are known to secrete phagocyte-activating type 1 cytokines like IFN-γ and TNF-α, which contribute to intracellular antimicrobial defense by activating macrophages (Kerksiek and Pamer, 1999; Schroder et al., 2004). Recent studies suggest that both CD4^+^ T cells and IFN-γ play critical roles in combating bacterial infections (Malley et al., 2005; Pilione and Harvill, 2006). The triple antigen fusion TFP846 contains three M.tb antigens: Rv3615c (Boesen et al., 1995), M.tb10.4 (Skjøt et al., 2000) and Rv2660c (Betts et al., 2002). Both Rv3615c and M.tb10.4 cover high density of T cell epitopes that promote strong T cell immune response, including functional T cell subsets secreting both IFN-γ and IL-2 (Millington et al., 2011). Rv2660c is stably expressed in the early and late stages of M.tb infection, and significantly enhances protective immunity characterized by a high proportion of multifunctional CD4+ T cells against M.tb infection in mice and cynomolgus macaques (Aagaard et al., 2011; Lin et al., 2012). Here, a strong cellular immune response was induced with a single dose of VSV-846, as shown by increased IFN-γ release, lymphocyte proliferation, and T cell cytotoxicity (Figures 3A–C).

Although similar bacterial loads were measured in VSV-846 and BCG immunized mice 6 weeks post-challenge, these became significantly different at long-term intervals, including 12 and 24 weeks post-challenge. This result differed from that reported by Roediger (Roediger et al., 2008), where VSVAg85A only conferred transient protecion from pulmonary M.tb challenge following single respiratory mucosal immunization. Considering that the same viral vector system was used, possible explanation is that the triple-antigen fusion TFP846 is more robust at inducing anti-TB immune responses as compared with using single M.tb antigens.

Memory T cells contribute to host defenses during a wide range of viral and intracellular bacterial infections (Harty et al., 2000). Both the effector memory T cells and the sustainable, high proliferative capacity of central memory T cells are important for a potentially successful TB vaccine (Andersen and Woodworth, 2014; Nunes-Alves et al., 2014). CD62L is a lymph node homing receptor that is down-regulated upon CTL activation (Ottenhoff and Kaufmann, 2012). CD44 is a surface protein required for lymphocyte extravasation to inflammatory sites and its up-regulation represents a marker for memory T cells (Harty et al., 2000). In this study, we observed that a large population of CD44^+^ and CD62L^Low^ memory T cells from isolated splenocytes 24 weeks post-vaccination. Although we observed much higher percentage of CD44^+^ and CD62L^Low^ memory T cells following VSV-846 immunization relative to those of BCG-immunized mice, we were unable to determine whether these memory T cells were generated from VSV-846 induction without antigen-specific tetramer staining. Previously, a VSV-based vaccine reportedly induced poly functional T cells proliferation that secrete activating cytokines, such as IL-2, IFN-γ and TNF-α, and contribute to antiviral defense (Wu et al., 2014). However, no study has shown the capability of vaccine-elicited cells secreting multiple cytokines during infection to develop into long-lived memory T cells exhibiting enhanced capability to control infection, as evidenced by the clinical failure of MVA85A (Ottenhoff and Kaufmann, 2012).

In conclusion, our results indicate that the VSV-based TB vaccine VSV-846 elicited robust cellular immune responses, as well as memory T cell responses, and it protects vaccinated mice against BCG infection over long-term periods of time following a needle-free single dose. These findings showed that use of VSV as an antigen-delivery vector is a potentially successful option for TB-vaccine development.

## Author contributions

CD and SX designed the study. MZ performed the experiments. MZ, CD, and SX interpreted the data. MZ, CD, and SX wrote the manuscript. All authors approved the final version of the paper.

## Funding

This work was supported by Major State Basic Research Development Program of China (2013CB530501, 2013CB531502), grants from the National Science & Technology Key Projects during the Twenty Five-Year Plan Period of China (2013ZX10003007), the National Natural Science Foundation of China (31470839, 81072428, 31270977), Priority Academic Program Development of Jiangsu Higher Education Institutions (PAPD), Jiangsu Provincial Innovative Research Team.

### Conflict of interest statement

The authors declare that the research was conducted in the absence of any commercial or financial relationships that could be construed as a potential conflict of interest.
